# The American Institute of Homœopathy

**Published:** 1884-03

**Authors:** P. P. Wells

**Affiliations:** Brooklyn, N. Y.


### HOMŒOPATHIC PHYSICIAN.

A MONTHLY JOURNAL OF MEDICAL SCIENCE.

"If our school ever gives up the strict inductive method of Hahnemann, we are lost, and deserve only to be mentioned as a caricature in the history of medicine."—CONSTANTINE HERING.

Vol. IV. MARCH, 1884. No. 3.

### "THE AMERICAN INSTITUTE OF HOMŒOPATHY."

P. P. WELLS, M. D., BROOKLYN, N. Y.

In 1844 a company of believers in and practitioners of what was understood to be a system of practical medicine, first given to the modern world in the *Organon of Homœopathic Medicine*, by Samuel Hahnemann, met in New York for the purpose of organizing an association for extension of a knowledge of this system and of the means it employed for the cure of the sick. The organization, then and there effected, received as the title by which it would be known in the history of this system that which stands as the title of this paper. This system, as understood by these gentlemen, was made up of well-defined elements, which may be grouped together under two heads—materia medica and therapeutics. All other branches of professional science were held by these organizers, in common with physicians of other schools of medicine of whatever name, just in the ratio in which these sciences had been mastered by these and other physicians. These were the same, and were alike taught by all who claimed to teach "scientific medicine." But the materia medica and therapeutics of the homœopathic school were unique. They were its own peculiar property, and wholly separate in their characteristics from what was called the materia medica and therapeutics of all other schools. So that when these physicians organized and took this name for the purpose of extending a knowledge of and confidence in the philosophy and practice of Homœopathy, it was a knowledge of this materia medica and of its right application for the cure of the sick (therapeutics) to which their action had reference. This body so then and there organized for this specific purpose, and for the protection of the community from the imposition of pretenders, still exists, and annually appears as a living body, doing work of a certain kind. The object of this paper will be to see, if we may, how far that work is in harmony with the original objectives of the Institute and in its nature tending to the furtherance of them.

If we look at the published organization of the Institute for work in the present year (1884) we shall find it to consist of fifteen bureaus or committees, to each of which is given a particular branch of science for its consideration, and on which each is expected to present a report of its work at the annual meeting of the Institute in June, 1884. Only five of these fifteen bureaus have subjects in any way related to Homœopathy other than the general relationship which every branch of science has to every other. Of the five which may be supposed to have a more possible relationship to the original objectives of the Institute, two of them, Clinical Medicine and Pœdology, have subjects assigned to each member, on which he is expected to write and send his paper to the June meeting. The subjects so assigned are certainly important, and properly studied and presented in the expected reports, as it is to be hoped they will be, can hardly fail of being interesting and perhaps profitable to the members of the body which will receive them. But it is noteworthy that of the twenty subjects so assigned not one of them need in its report have any reference to Homœopathy at all. Clinical Medicine has to deal with "Indiscretions of Life and their Relation to Disease"—an important subject certainly, and well worth careful study, but he has sharp vision who can see any Homœopathy in it. Pœdology has "Infantile Dentition and Its Incident Diseases"—not a subject of small importance; but where is the objective of the Institute to be brought in, unless it may be supposed included in the expression used in assigning the second paper—"Causes and Cures"? And is this possible inference of a homœopathic relation all we can find of Homœopathy in the proposed work of the Institute for 1884? Is this the best showing it has of its present ways of carrying out the proposed work of this body, as declared by its originators? Is the relation of this Institute to therapeutics as founded on law for the year 1884 expressed, and only expressed, in this faint inference? If so, then are we not justified in charging the body with dishonoring the memory of its founders, whom they are in the present supposed to represent, and with treating the great objectives of these founders with contempt?

But is not the case with the Bureau of Materia Medica, the other branch of the objective of the Institute, to be better? Is there not reason for hope of something in their labors which may strengthen the interests of our philosophy and practice with the profession or the public? Let us see if we have ground for such hope, and if so, on what is that hope founded?

First, let us remember that our materia medica is one of the essentials of our practice, and is one of the chief elements in all practice founded on law. It lies as one of its chief foundation stones, so that a body organized for the advancement of this practice might be supposed to be, more than others, jealous for its reputation and honor. The interests of this fundamental element in all homœopathic practice were committed to a bureau consisting of nine members. We have known something of some of these members, and something of their work in relation to our materia medica heretofore, and what we know of this is not assuring of hopes of good for the near future as the outcome of their labors for the present year. If we understand the manner of construction of this bureau, the chairman is appointed by the President of the Institute, and then the chairman selects his associates, and the bureau so made up is to give impulse for good or evil to this important branch of our science for the coming year. In forecasting the probable character of the work of this bureau, it is natural and necessary to begin with the character of its constituent members so far as this is known. And first, of its chairman: He has made himself conspicuously and sufficiently known for some years by his efforts to destroy the confidence of whoever might confide in him in our materia medica, and to this end has shown himself its busy and constant slanderer and detractor, and the only imaginable reason which we can give for his not having done it greater damage is the fact, also conspicuous, that his powers are rather limited. No one will deny that he has done all he could, and at all times, to destroy public and professional acceptance of this inestimable and exhaustless treasure of the fruits of the labors of those who in the past have been our greatest, best, and most successful cultivators of homœopathic science and practice. That the destruction wrought has been small is not because his efforts have not been indefatigable, but because his powers were small. The fact of this member's labors in the interests of destruction could not but have been well known to the presiding officer of the Institute by whom he was appointed to this chairmanship. If his history of these labors was not the only incentive to his appointment, we cannot conceive of any other. And if this appointment be received as evidence of the truth of the suspicion, already expressed, that the one great endeavor of the Institute is in these last times to accomplish its own destruction, who shall blame those who thus receive it?

And then this man, so thoroughly pervaded by apparent hatred of our materia medica and so constant in his endeavors to slander it, had the choice of his associates, and, of course, would look first for those, if any could be found, who in their regard for the interests of the subject to be committed to their care were of like mind with himself. And there were not wanting those who had been, like himself, employed in endeavors to sap confidence in our materia medica, and he called the most conspicuous of them to his side; and now we have only to await the result of their united efforts, probably, to destroy. The outlook does not appear as boding any good to the Institute or to science. We do not, therefore, anticipate any very great damage as the result of this combination. The Institute may be disgraced, as it has been in the past at times, by the folly, ignorance, or indiscretion of other members, but there is another history than that made by the present members of our bureau, which has so demonstrated the truth, *reliability*, and value of our materia medica that the detractions and "observations," so called, microscopes and all, will ultimately be found wholly powerless for evil before its long and bright career of successes. The members of our bureau have said, and notably its chairman—"Our materia medica pura is impure, and should be weeded out," and he seems to regard himself as just the man to do the weeding. But he has not done it. He has not even pointed out the weeds or shown us what they are—to say nothing of the absence of all proof that these so-called weeds are indeed what they allege them to be. In the absence of such proof they—the bureau—may as well understand our best men do not believe a word they say on this subject, but go on as if this slander has not been spoken, and use the materia medica with confidence, and achieve cures therewith the same since as before the utterances of these would-be oracles. These cures, each and all, testify against these detractors, and demonstrate at least the rashness of their utterances.

If it be said of this chairman and others who, like him, have endeavored to defame our materia medica that it is only its errors of which they intended to speak and that it is only these they would have removed, we reply they have not proved the existence of the errors which this explanation assumes them to have antagonized; nor in aught that has come to our knowledge have they shown any fitness to sit in judgment on the facts of our materia medica and to decide this one or that to be true or false. The work would certainly be found most difficult, even by him who by largest knowledge might be regarded as most competent for the task. It is certain this chairman has shown no fitness for it by any act of his which has come to our knowledge, while, if we are not mistaken, his urgent ambition to undertake the work goes far to demonstrate his entire unfitness for it. It more than suggests an utter absence of all perception of its difficulties and responsibilities. This eagerness to revise the work of those who were so greatly his superiors indicates neither modesty nor discretion. He who would undertake this great work should be possessed of both.

And, further, it may be replied to those who have and do still habitually disparage our materia medica, that the great masters who by their wonderful record of healings found their armamentarium in this alleged impure work. They had no other. They found it equal to all their needs. They were never found abusing it or prating of its impurities and errors. They knew how to use it, and used it; and in its use—whatever its errors—they, and they only, made a record which has given to the system of medicine they practiced a world-wide acceptance. In view of this fact, we are strongly impressed with the thought that one important difference between those who then used our materia medica and those others who are chiefly known by their abuse of it, is that they on the one side *knew how to use it*, while those on the other do not.

It may be further remarked of this bureau and its probable and perhaps expected work, that if the object of its appointment —in its present character—were to give aid and countenance to previous destructive influence and effort, the prospect of success in this is too apparent to be pleasant in contemplation. If the judgment were that in furthering the destruction of Homœopathy —as some think the late history of the Institute intended to do— the first and most potent step would be the destruction of our materia medica, or, what would be a near equivalent to this, destroy confidence in its purity and truth, the judgment was evidently a sound one. There can be no doubt that, our materia medica gone, and Homœopathy is gone. Practically its existence would become impossible. This is at once evident when it is remembered that practical Homœopathy rests on its materia medica; so that, destroy the foundation, and the superstructure will topple to the ground, is not only a sound judgment, but, in the assignment of the interests of our materia medica to this bureau, do we not recognize a shrewd device for the destruction of our system of practical medicine, which some say they have recognized as the end of the policy of the Institute for the last few years? Some have been so bold in their desire to wipe out all which is characteristic of our school that they have not hesitated to propose striking out the word "Homœopathy" from the name of our Institute. It is easy of conception that he who proposed this innovation reasoned somewhat in this wise when concocting his radical proposition: "Why not let this go, when all else characteristic of Homœopathy is so nearly gone—swallowed up in endeavors to emulate and imitate the teachings and doings of old-school physic—and then we can all become good doctors and good fellows together?" If he so reasoned in the circumstances, who is there who can give a good reason why not? Is it not possible, when he sees the constitution of our Bureau of Materia Medica and the exclusion so largely (almost entirely) of Homœopathy from the prepared programme for 1884 in favor of so many only related sciences or branches of science, he may be tempted to renew his proposition? and if he does, who will say he has not a reasonable promise of success in the arrangement for 1884?

We have alluded, and not approvingly, to the exclusion of Homœopathy in favor of other branches of science, not because a study and knowledge of any one of them is unimportant—far from this. But it has seemed sad and wrong that that more important science of therapeutics, founded on God's law—in the interests of which and for the inculcation and defense of which this Institute was created—should have been made to give place to these less important branches of knowledge only incidentally related to this greater, and so to furnish a new impulse in the direction of the alleged movement, for some time said to have been determined on and operative, for the destruction of that for the preservation, continuance, and enlargement of which this Institute was created. That it should now seem, by indifference or intent, to allow this infinitely more important science to be swallowed up in a greater interest in other knowledges, which, whatever their importance, are so far beneath that other divine law of healing, is, to say the least, an additional and wholly unneeded evidence of human perverseness or stupidity.

